# The Impact of Triangle Hierarchical Management on Self-Management Behavior and Quality of Survival in Parkinson's Patients

**DOI:** 10.3389/fsurg.2022.878477

**Published:** 2022-04-15

**Authors:** Yahua Zeng, Jianghua Huang, Xuan Tang, Ting Wang, Shuangqin Chen

**Affiliations:** ^1^The First Affiliated Hospital, Department of Rehabilitation, Hengyang Medical School, University of South China, Hengyang, China; ^2^The First Affiliated Hospital, Department of Neurology, Hengyang Medical School, University of South China, Hengyang, China

**Keywords:** Parkinson's, Triangle, tiered management, self-management behavior, quality of survival

## Abstract

**Objective:**

To investigate the effect of Triangle tiered and graded management on the self-management behavior and quality of survival of Parkinson's Disease (PD) patients.

**Methods:**

Eighty ambulatory PD patients admitted to the neurology outpatient clinic of our hospital from June 2020 to January 2021 were selected for the study. Eighty patients were divided into 40 cases each in the test group and the control group using the random number table method. Patients in the control group were given conventional treatment and care, while in the test group, Triangle hierarchical management was applied on the basis of the control group. Non-motor symptoms [assessed by the Montreal Cognitive Inventory (MoCA), the Scale for Outcomes in PD for Autonomic Symptoms disability Scale (SCOPA-DS) and the Nocturnal Scale (SCOPA-NS)], motor symptoms [assessed by the Functional Gait Assessment (FGA), the Modified Ashworth Scale, and the Unified Parkinson's Disease Rating Scale (UPDRS-III)], quality of life (assessed by Barthel Index), medication adherence (self-administered medication adherence questionnaire), quality of survival (assessed by the 39-item Parkinson's Disease Quality of Survival Questionnaire, PDQ-39), and self-management effectiveness (assessed by the Chronic Disease Self-Efficacy Scale, symptom management and disease co-management) were compared between the two groups before and after the intervention. The two groups were also observed for satisfaction with care.

**Results:**

After the intervention, the MoCA score, FGA score, Barthel Index, Medication adherence and all scores of self-management effectiveness were significantly higher in the test group than in the control group (*P* < 0.05); the SCOPA-DS score, SCOPA-NS score, Ashworth score, UPDRS-III score and PDQ-39 score were significantly lower than in the control group (*P* < 0.05). Satisfaction with nursing care was significantly higher in the test group than in the control group (*P* < 0.05).

**Conclusion:**

The application of Triangle's tiered and graded management to the home care of ambulatory PD patients was effective in improving their non-motor and motor symptoms, their ability to perform daily activities, medication adherence and self-management effectiveness, and their overall survival outcome.

## Preface

With the accelerated aging of the population, Parkinson's disease (PD) has become the second most serious progressive neurodegenerative disease after dementia that affects the elderly worldwide (1, 2). The motor and non-motor symptoms of PD can cause a range of functional impairments of varying severity, seriously jeopardizing patients' physical and mental health and quality of life, and increasing the incidence of accidental risk and mortality (3). PD is not yet completely curable, so clinical treatment is primarily aimed at slowing the disease process, improving functional impairment, reducing the risk of complications and improving survival. However, the complexity of pharmacological regimens and fear of side effects have led to a decline in compliance, while the prolonged, irreversible, slow-onset nature of the disease and the lack of family support have severely undermined patients' self-confidence, resulting in a majority of patients being unable to sustain functional rehabilitation and thus exacerbating the disease's progression (4, 5). Therefore, it has become a major concern for the community to improve the effectiveness of pharmacological and rehabilitative treatments to improve the clinical outcome of PD patients.

Recent studies (6–8) have shown that as the concept and content of nursing services continue to develop, the nursing management model, as an effective complement to medical services, can effectively enhance the treatment and rehabilitation of patients with various chronic diseases. It has a positive effect on improving patients' prognosis, preventing disease recurrence, improving quality of life and reducing the burden on patients and their families. The Triangle Chronic Disease Tiered Management Model was developed by Kaiser Permanente, a large managed care organization in California, and has been used since 2002 to manage the care of patients with chronic diseases (9, 10). The model divides patients into high-risk, moderate-risk, and stable tiers, and then provides specialized medical care proportional to the needs of each tier, creating a pyramidal tiered management model that is more economical and The model is a pyramidal hierarchical management model that treats patients more economically and effectively (11). This study refers to the Triangle Chronic Disease Stratified Management Model (12) and combines it with the Hoehn-Yahr classification of PD (13) to construct stratified and graded management criteria that are consistent with ambulatory PD patients. Ambulatory PD patients were classified as high-risk patients, moderate-risk patients and stable patients for management at three levels, and three levels of care were provided for case care, self-management and disease management. The aim is to improve treatment adherence, self-management and quality of survival for PD patients.

## Materials and Methods

### Study Population

Eighty ambulatory PD patients admitted to the neurology outpatient clinic of our hospital from June 2020 to January 2021 were selected for the study.

### Inclusion and Exclusion Criteria

Inclusion criteria: (i) patients who met the new clinical diagnostic criteria for PD established by the International Movement Disorders Society (MDS) in 2015 (14); (ii) patients aged 18–70 years old; (iv) patients who were in the stable stage of the disease, were mentally alert, had no language communication impairment and had the ability to understand; (v) patients and family members who gave informed consent and voluntarily participate in this study.

Exclusion criteria: (i) Patients who did not agree to participate in the study. (ii) Patients who were not in their right mind, had difficulty in verbal communication and had cognitive impairment. (iii) Patients without the ability to understand. (iv) Patients who died midway or withdrew from the intervention trial. (v) Patients with severe depression and severe anxiety.

### Sample Size Calculation

The sample size was estimated based on the formula for the sample size required for comparison of the means of two samples, based on a two-sided α = 0.05, 1–β = 0.90 and assuming δ/σ = 0.80, and the sample size was derived from the attached table as 34 cases per group. However, considering the possible sample attrition during the study, the sample size was expanded by 20% from the original one, and the final sample size was determined to be 40 cases per group, with a total of 80 cases. A random number table was used to divide the 80 patients into a test group and a control group of 40 patients each. Patients in the control group were given conventional treatment and care, while the test group was managed by Triangle stratification on the basis of the control group, and both groups were intervened and followed up for 12 months.

### Intervention Methods

The baseline survey included general information on gender, age, marital status, duration of illness, financial income and severity of illness, the Parkinson's Disease Rating Scale Part III (UPDRS-III), the Medication Adherence Scale, the 39-item Parkinson's Disease Quality of Life Questionnaire (PDQ-39) and the Chronic Disease Self-Efficacy Scale.

Control group: Patients were given the usual medication, diet, sleep, rehabilitation exercise instruction and psychological care. The duration of intervention was 12 months, with monthly telephone follow-ups and monthly outpatient follow-ups in the 3rd, 6th and 12th months.

Test group: The Triangle stratification and grading management model was used to stratify ambulatory PD patients in the following ways. i. At the time of consultation, patients were classified into a smooth stratum (Hoehn-Yahr classification of 1 to 1.5), an intermediate risk stratum (Hoehn-Yahr classification of 2 to 3) and a high risk stratum (Hoehn-Yahr classification of 4 to 5) according to the baseline findings and with reference to Triangle stratification and Hoehn-Yahr classification criteria for PD. Establish follow-up files for ambulatory PD patients at each tier. The files report the patient's general information, contact information, clinical manifestations and examination results, etc. The files for the smooth, medium-risk and high-risk tiers are marked in green, orange and red, respectively. The follow-up methods we adopt are telephone follow-up, outpatient follow-up, home follow-up, WeChat and WeChat platform, etc. The graded management criteria for patients are specified in Table 1. iii. Tier flow: Patients are reassessed once after the intervention in 1, 3, 6, and 12 months according to the tier criteria to determine the number of tier flow instances, as well as file reorganization and management into the new tier criteria.

**Table 1:** Stratified and graded management criteria for ambulatory PD patients.

**Criteria for patient stratification**	**Grading management standards**
Smooth stratum Grade 1: Unilateral limb disease. Grade 1.5:unilateral limb combined with trunk (axial) symptoms.	Tertiary follow-up care requirements: (1) Outpatient follow-up once every 3 months; (2) Telephone or other follow-up once every month; (3) Daily exercise training, no more than 45 min each time, 3 times/day; (4) Population health education: mainly conventional education (e.g., through books, videos, audio-visual materials, etc.), encouraging patients to participate in group activities; encouraging patients to present themselves and participate in the education of other patients, giving them the opportunity to serve as role models opportunities. (5) Follow-up: telephone follow-up.
Intermediate risk stratum Grade 2: bilateral limb symptoms but no balance disturbance. Grade 2.5: Mild bilateral symptoms with recovery from backward pull test. Grade 3: Mild to moderate bilateral symptoms, unable to recover from pull-back test, unstable posture, slower turning, many functional limitations but patient is able to care for himself/herself.	Secondary follow-up care requirements: (1) Outpatient follow-up once every 2 months; (2) Telephone or other means of follow-up once every half month; (3) Daily exercise training of no more than 40 min each time, two times/day; (4) Point follow-up care: health education is based on group education, for patients who cannot participate in group education, individual education can be adopted. (5) Follow-up methods: telephone follow-up, outpatient follow-up, WeChat, WeChat platform, etc.
High risk stratum Grade 4: Severely disabled, able to stand and walk without assistance. Grade 5: Wheelchair-bound or bedridden, totally dependent on others for assistance.	Primary follow-up care requirements: (1) Outpatient follow-up once every 1 month; (2) Telephone or other follow-up once every 7 days; (3) Daily exercise training not exceeding 20 min each time, 2 times/day; (4) Case follow-up care: according to the patient's condition, individualized care plans are formulated and individualized, targeted health education is implemented. (5) Follow-up methods: telephone follow-up, outpatient follow-up, home follow-up, WeChat, WeChat platform, etc.

### Evaluation Indicators

Non-motor symptoms: The Montreal Cognitive Inventory (MoCA), the Scale for Outcomes in PD for Autonomic Symptoms disability Scale (SCOPA-DS) and the Nocturnal Scale (SCOPA-NS) were used to assess the pre and post intervention. The MoCA scale consists of 8 dimensions with a total score of 30, with higher scores indicating better cognitive function. scores range from 0 to 15 for the SCOPA-NS and from 0 to 18 for the SCOPA-DS, with lower scores indicating better sleep.

Motor symptoms: Functional Gait Assessment (FGA), modified Ashworth Scale, Unified Parkinson's Disease Rating Scale Part III (UPDRS-III) were assessed before and after the intervention. The FGA assessed the patient's functional gait from items 1–10 of the FGA, and each item was scored on a 4-point scale from 0 to 3 out of 30, with higher scores indicated better balance and walking ability. A modified version of Ashworth was used to assess the patient's muscle tone, and scores from 0 to 4 were assigned from normal muscle tone to stiffness during movement of the affected area. UPDRS-III included 14 items with a total score of 0 to 70, with higher scores indicated poorer motor function.

Ability to perform activities of daily living: A comprehensive evaluation using the Barthel Index rating of 10 items such as eating, grooming, continence control and bed and chair transfer, each with a score of 10, for a total score of 100. A higher score indicates that the patient needs less help and is less dependent.

Medication adherence: A self-made PD medication questionnaire was used to evaluate the medication compliance of the two groups of patients, and the internal consistency of the questionnaire was 0.813. The total score of the questionnaire was 100 points, with a score of 86–100 indicated complete compliance, 70–85 indicated partial compliance, and ≤ 69 indicated non-compliance.

Quality of survival: The 39-item Parkinson's Disease Quality of Survival Questionnaire (PDQ-39) was used to assess the quality of survival. The scale consists of 39 items in 8 dimensions, each with five health levels (0 to 4), and the sum of the scores for each item was converted to a percentage, with higher total scores indicating lower quality of survival.

Self-management efficacy: The Chronic Disease Self-Efficacy Scale was used to assess the patient's self-management efficacy, which consists of 6 items in 2 dimensions, namely symptom management self-efficacy and disease co-management self-efficacy. The scale is rated on a scale of 1~10, with higher scores indicating higher self-management efficacy, in which the total score <4 points is regarded as low level; 4 ~ 7.9 points are regarded as medium level; ≥8 points are regarded as high level.

Nursing satisfaction: The nursing satisfaction questionnaire for ambulatory Parkinson's disease patients developed by the nursing department of our hospital was used to investigate the satisfaction of the two groups of patients with nursing services, which were divided into extremely satisfied, generally satisfied and unsatisfied, and the satisfaction rate = extremely satisfied rate + general satisfaction rate.

### Statistical Methods

SPSS 22. 0 software was used for data analysis. The statistical data were expressed as percentages using the χ^2^ test, and the measurement data were expressed as $\bar{x}$±s using the t test. The difference was considered statistically significant at *P* < 0.05

## Results

### Comparison of Baseline Information Between the Two Groups

The differences in gender, mean age, duration of disease, monthly personal income, marital status and Hoehn-Yahr classification among the different groups of ambulatory PD patients were not statistically significant (*p* > 0.05) and subsequent comparisons could be made. As shown in Table 2.

**Table 2:** Comparison of baseline information between the two groups (%, $\bar{x}$ ± s).

**Items**		**Control group** **(*n* = 40)**	**Test group** **(*n* = 40)**	**t or χ^2^ value**	***P-*** **value**
Gender	Male	19 (47.50)	17 (42.50)	0.202	0.653
	Female	21 (52.50)	23 (57.50)		
Mean age (years)	64.21 ± 8.74	65.20 ± 8.17	0.523	0.602	
Duration of illness (years)	5.23 ± 2.46	4.87 ± 1.69	0.763	0.448	
Personal monthly income (yuan)	0~999	5 (12.50)	7 (17.50)	1.189	0.756
	1,000–2,999	17 (42.50)	19 (47.50)		
	3,000–4,999	10 (25.00)	9 (22.50)		
	>5,000	8 (20.00)	5 (12.50)		
Marital status	Unmarried	3 (7.50)	5 (12.50)	1.900	0.387
	Married	29 (72.50)	31 (77.50)		
	Widowed or divorced	8 (20.00)	4 (10.00)		
Hoehn-Yahr grading	1–2	7 (17.50)	9 (22.50)	1.293	0.524
	2.5–3	30 (75.00)	27 (67.50)		
	4–5	3 (7.50)	4 (10.00)		

### Comparison of Non-motor Symptoms

**Figure 1:**
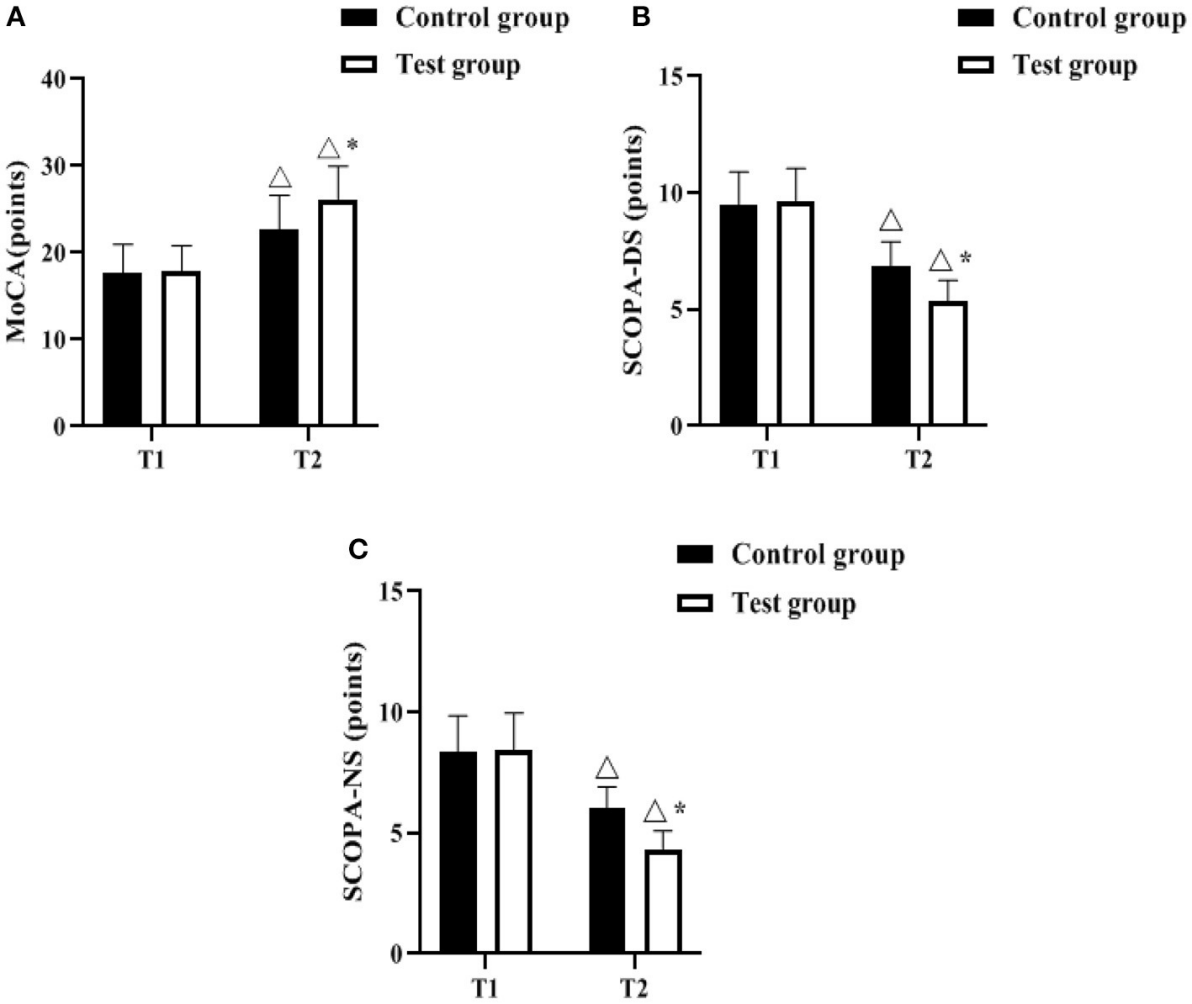
Comparison of non-motor symptoms (*n*, $\bar{x}$± s). **(A–C)** indicate the MoCA, SCOPA-DS, and SCOPA-NS scales, respectively. Icon Δ indicates the difference between T2 period and T1 period between the same groups, *P* < 0.05. Icon * indicates the difference between the two groups in T2 period, *P* < 0.05.

The MoCA, SCOPA-DS and SCOPA-NS scales were used to assess the degree of non-motor symptoms of the patients before and after the management intervention. As shown in Figures 1A–C, the differences in the scores of MoCA, SCOPA-DS, and SCOPA-NS between the control group and the test group before the intervention (T1) were not statistically significant (*P* > 0.05). 12 months after the Triangle stratified management intervention (T2), MoCA scores increased in both groups compared to T1, and were higher in the test group (*P* < 0.05); SCOPA- DS and SCOPA-NS scores decreased in both groups compared to T1, and were lower in the test group (*P* < 0.05).

### Comparison of Movement Symptoms

**Figure 2:**
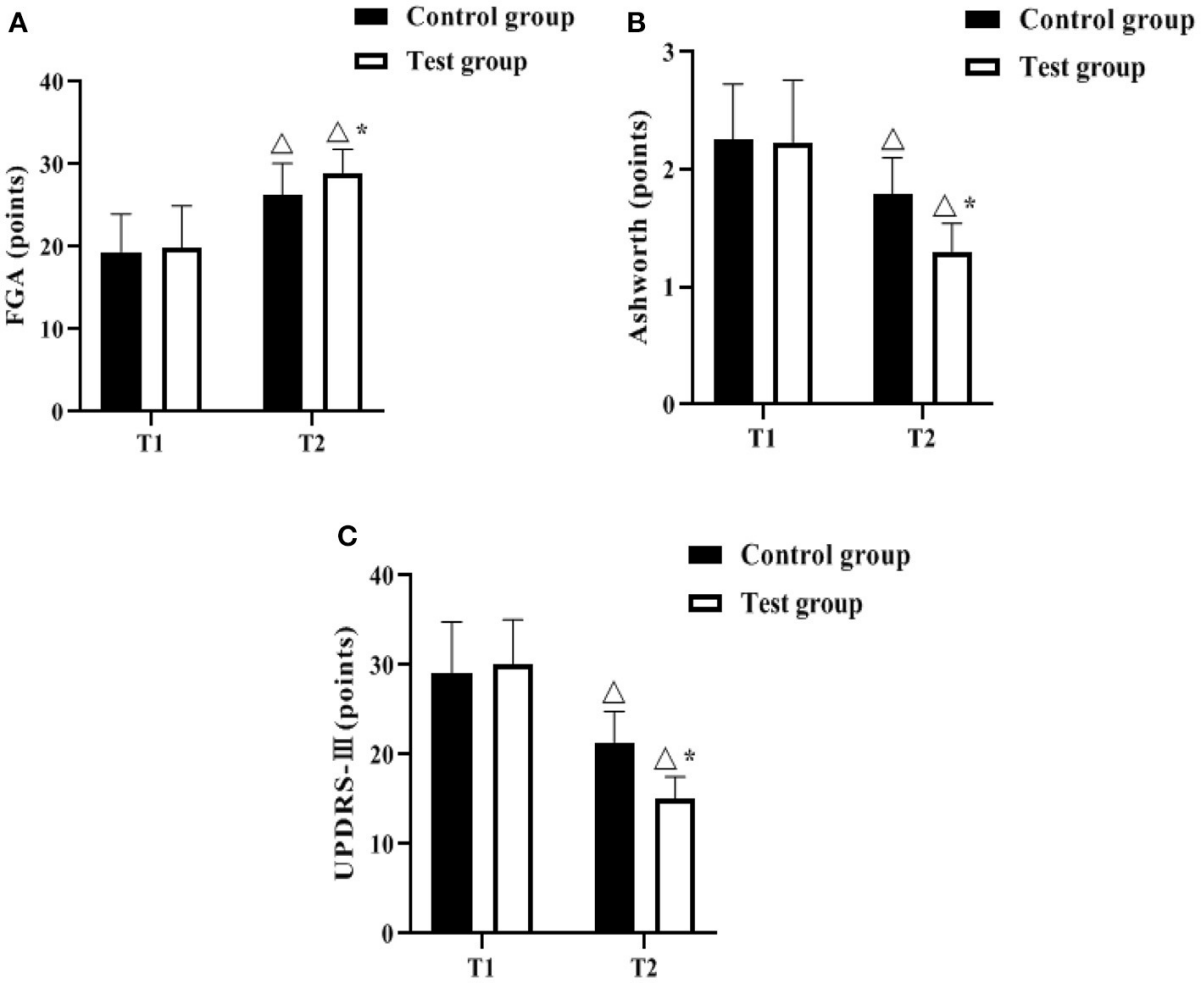
Comparison of movement symptoms (*n*, $\bar{x}$ ± s). **(A–C)** indicate the FGA, Ashworth and UPDRS-III scales, respectively. Icon Δ indicates the difference between T2 period and T1 period between the same groups, *P* < 0.05. Icon * indicates the difference between the two groups in T2 period, *P* < 0.05.

The FGA, Ashworth and UPDRS-III scales were used to assess the degree of motor symptoms of the patients before and after the management intervention. As shown in Figures 2A–C, there was no difference between the control group and the test group in comparing the scores of FGA, Ashworth, and UPDRS-III at T1 (*P* > 0.05). The FGA scores of both groups increased at T2 compared with T1, and were higher in the test group (*P* < 0.05); the Ashworth and UPDRS-III scores of both groups decreased compared with T1, and were lower in the test group (*P* < 0.05).

### Comparison of Barthel Index, Medication Adherence, PDQ-39 Scores

**Figure 3:**
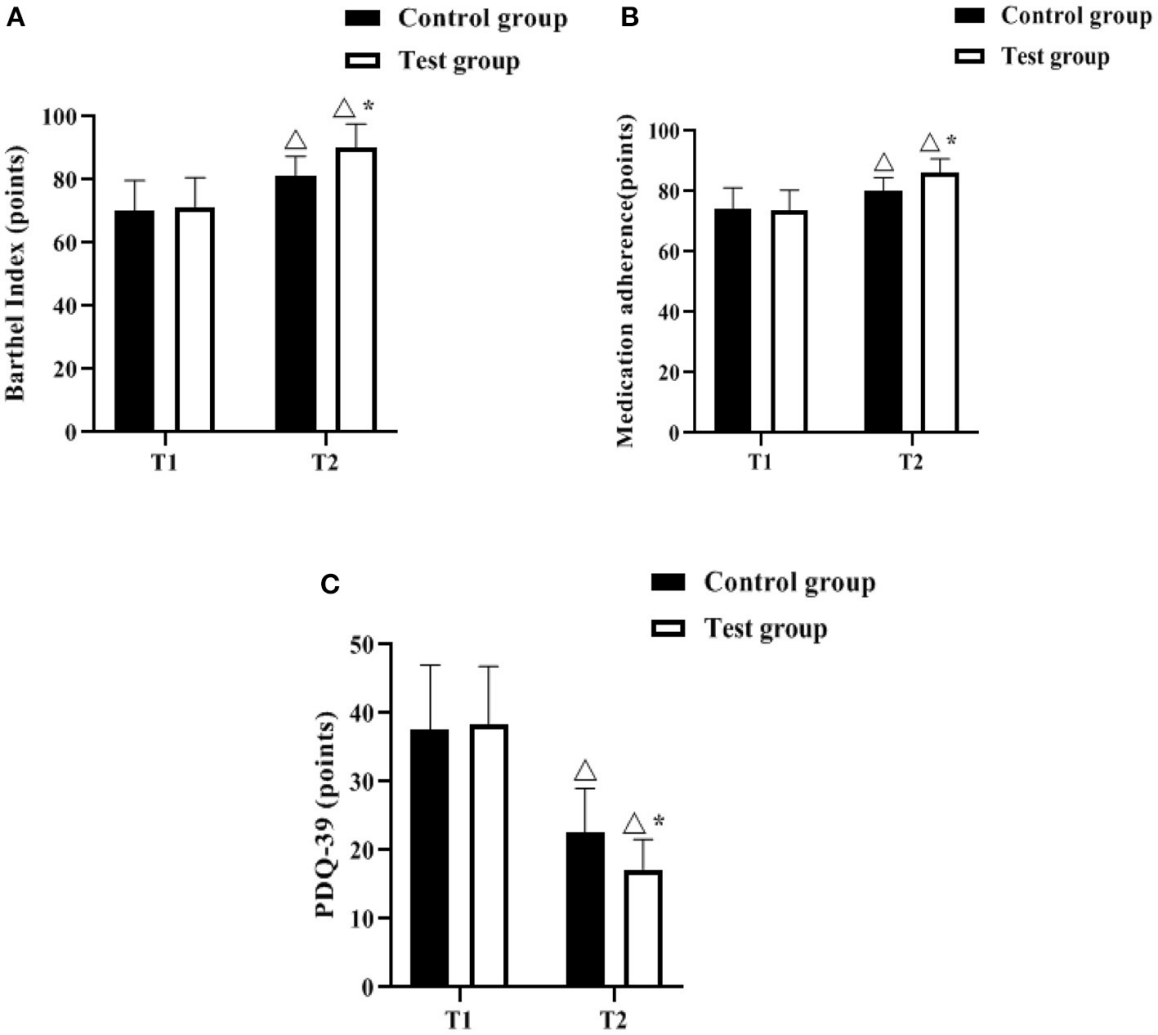
Comparison of barthel index, medication adherence, PDQ-39 scores (*n*, $\bar{x}$ ± s). **(A–C)** indicate the Barthel Index, Medication adherence and PDQ-39 scales, respectively. Icon Δ indicates the difference between T2 period and T1 period between the same groups, *P* < 0.05. Icon * indicates the difference between the two groups in T2 period, *P* < 0.05.

The Barthel Index, Medication adherence and PDQ-39 scales were used to assess the ability to perform daily activities, medication compliance and quality of life before and after the management intervention, respectively. As shown in Figures 3A–C, there was no difference in Barthel index, medication adherence, and PDQ-39 scores at T1 between the control and experimental groups (*P* > 0.05). The Barthel Index and Medication adherence scores increased in both groups at T2 compared to T1, with the test group having a higher score (*p* < 0.05); the PDQ-39 scores decreased in both groups compared to T1, with the test group having a lower score (*p* < 0.05).

### Comparison of Self-Management Effectiveness Scores

**Figure 4:**
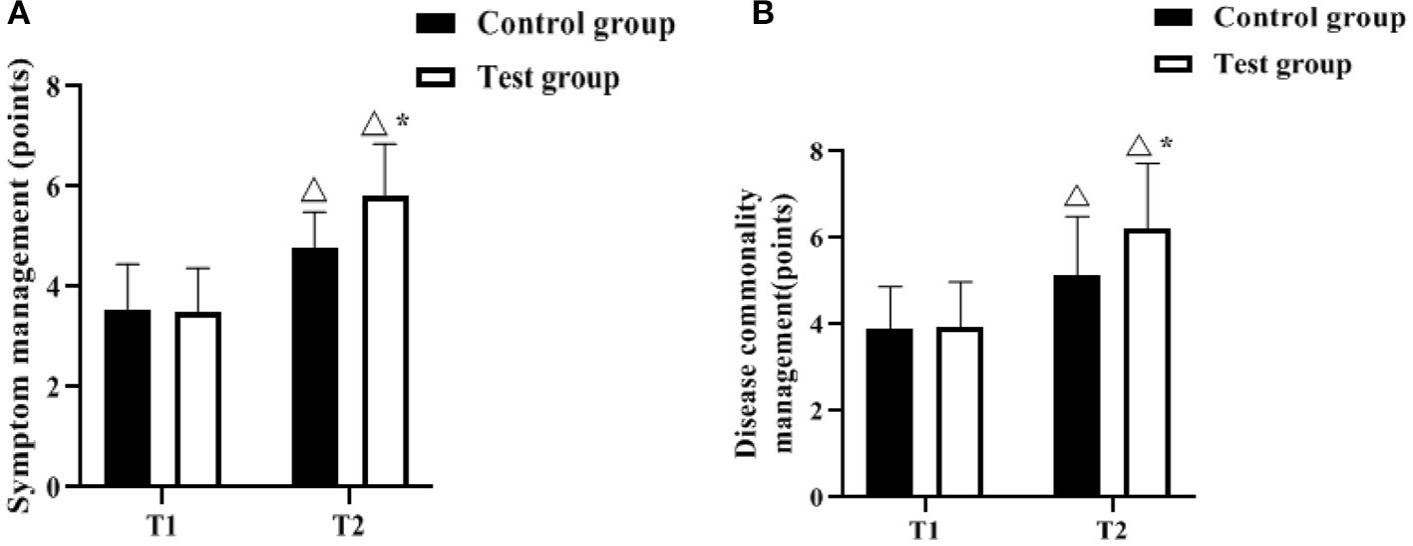
Comparison of self-management effectiveness scores (*n*, $\bar{x}$ ± s). **(A,B)** indicate the symptom management, disease commonality management scores, respectively. Icon Δ indicates the difference between T2 period and T1 period between the same groups, *P* < 0.05. Icon * indicates the difference between the two groups in T2 period, *P* < 0.05.

The Chronic Disease Self-Efficacy Scale was used to assess the patients' self-management effectiveness before and after the management intervention. As shown in Figures 4A,B, there was no difference in symptom management and disease co-management scores between the control and test groups at T1 (*P* > 0.05). The symptom management and disease commonality management scores increased in both groups at T2 compared to T1, and were higher in the test group (*p* < 0.05).

### Nursing Satisfaction

**Figure 5:**
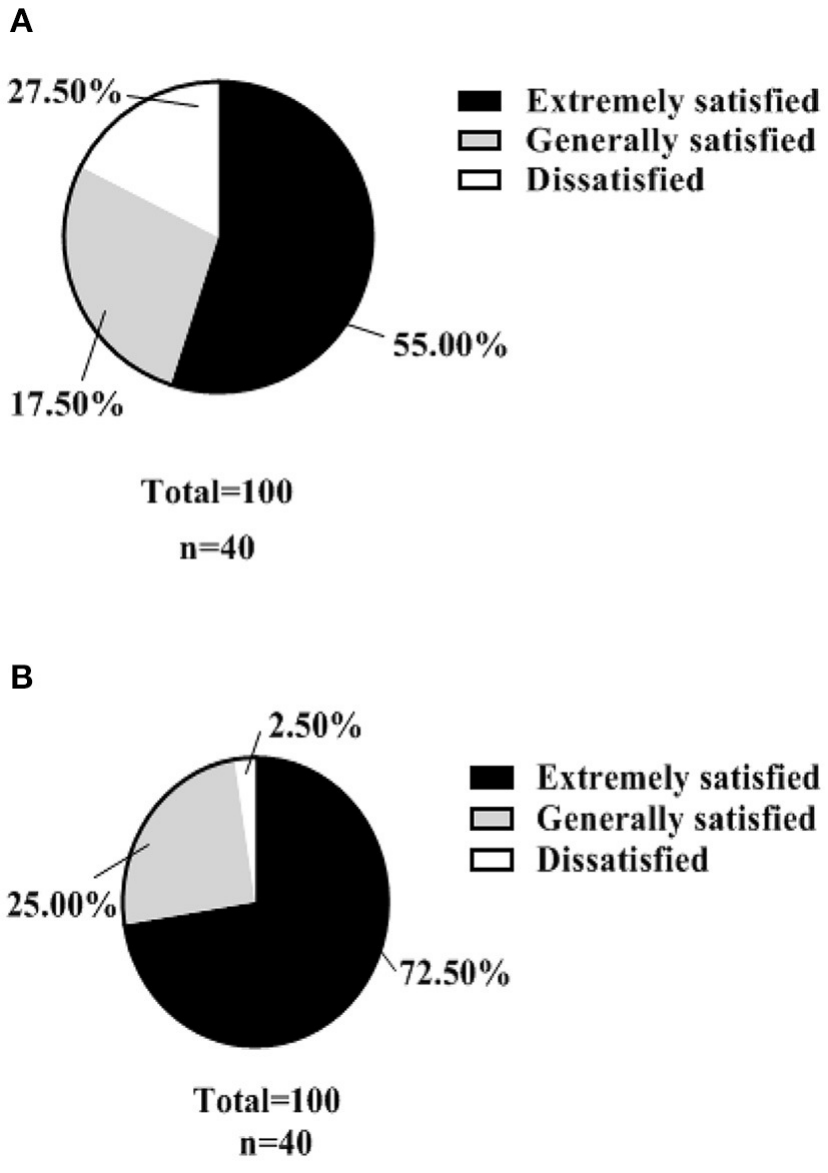
Nursing satisfaction (*n*, %). **(A)** shows the satisfaction rate of patients in the control group with the management of their care. **(B)** shows the satisfaction rate of patients in the test group with the nursing management.

A hospital-made questionnaire was used to assess patients' satisfaction with nursing management. In the control group, the number of patients in the three levels of “extremely satisfied,” “generally satisfied” and “dissatisfied” were 22, 11, and 7, respectively, with a satisfaction rate of 82.50% (33/40). In the test group, there were 29, 10 and 1 cases of “extremely satisfied,” “moderately satisfied” and “dissatisfied”, respectively, with a satisfaction rate of 97.50% (39/40). As shown in Figure 5.

## Discussion

PD is a typical example of a functional neurosurgical disorder that is a movement disorder with genetic, age-related and environmental causes and a pathology that results from the deformation and loss of pigment-containing neurons (15, 16). It is characterized clinically by slowly progressive movement disorders (resting tremor, bradykinesia, bradykinesia and postural gait abnormalities) and, as the disease progresses, by intellectual disability in advanced stages (17). As research into the pathophysiology of PD progressed, it was discovered that PD pathology can involve the peripheral nervous system and cerebral cortex, leading to non-motor symptoms such as anxiety, depression, sleep disturbances and cognitive changes, which are easily overlooked (18). In the early stages of PD, drug treatments such as dopamine preparations are effective in improving symptoms such as tremor and motor fluctuations, but as the disease progresses, the effectiveness of clinical interventions decreases and there are many problems such as adverse effects and drug interactions (19, 20). In addition to the prolonged duration of the disease, the patient's social and life skills are severely diminished, which not only affects the patient's quality of life, but also places a burden on the family and society, so the implementation of effective nursing interventions for patients based on clinical treatment has a positive impact on improving motor and non-motor symptoms (21).

The Triangle Chronic Disease Stratified Management Model suggests that different populations need to be identified and managed at the correct level of care for different conditions, increasing the effectiveness of management while reducing overall costs (22, 23). In this study, we envisage and attempt to develop a tiered and graded management model based on the Triagle Chronic Disease Tiered Management Model suitable for ambulatory PD patients and apply it to the management of ambulatory PD patients and the practice of tiered and graded management of ambulatory PD patients. The results of this study showed that there was a significant improvement in both motor and non-motor symptoms in both groups after 12 months of management intervention, with the test group outperforming the control group (*p* < 0.05). This suggests that Triangle's graded management significantly improved cognitive function, sleep quality and limb movement, mainly due to the fact that the test group developed a personalized exercise programme and follow-up programme based on the patients' graded condition, which helped to improve the patients' muscle and neurological functional limitations. In addition, the patient's confidence and adherence to treatment were enhanced by tailor-made care and individualized health education, which helped to improve non-motor symptoms such as cognitive function and sleep disturbance, as well as the recovery of social function (24, 25). The results also showed that Triangle stratified management significantly improved patients' activities of daily living, medication adherence, quality of life and self-management effectiveness, as evidenced by significantly higher Barthel Index, Medication adherence and Chronic Disease Self-Efficacy Scale scores in the test group than in the control group and before the intervention, and significantly lower PDQ-39 scores than in the control group and before the intervention (*P* < 0.05). Simplified personalized medication regimens, self-monitoring of symptoms, medication behavior management strategies, cognitive interventions, changes in dosing regimens, emotional management and reduced financial burdens can all contribute to improved medication adherence and self-management effectiveness in PD patients, thereby improving their quality of life and ability to perform daily activities (26, 27). In addition, the results of the nursing satisfaction survey showed that the test group was significantly more satisfied with the nursing management work than the control group, suggesting that Triangle's tiered and graded management can meet the needs of patients and their families for nursing services to a certain extent, with high acceptability, which is conducive to promoting patients' recovery.

In summary, Triangle's tiered and graded management applied to the home care of ambulatory PD patients was effective in improving their non-motor and motor symptoms, improving their ability to perform daily living activities, medication adherence and self-management effectiveness, and improving the overall survival of patients.

## Data Availability Statement

The original contributions presented in the study are included in the article/supplementary material, further inquiries can be directed to the corresponding author/s.

## Ethics Statement

The studies involving human participants were reviewed and approved by the Ethics Committee of Hengyang Medical School, University of South China. The patients/participants provided their written informed consent to participate in this study.

## Author Contributions

YZ is the mainly responsible for the writing of the article. JH is mainly responsible for research design. XT and TW are mainly responsible for data analysis. SC is responsible for the guidance of the entire research. All authors listed have made a substantial, direct, and intellectual contribution to the work and approved it for publication.

## Funding

This study was funded by the Hunan Provincial Health and Wellness Commission (No. 202214014506).

## Conflict of Interest

The authors declare that the research was conducted in the absence of any commercial or financial relationships that could be construed as a potential conflict of interest. The reviewer YZ declared a shared parent affiliation with the authors to the handling editor at the time of review.

## Publisher's Note

All claims expressed in this article are solely those of the authors and do not necessarily represent those of their affiliated organizations, or those of the publisher, the editors and the reviewers. Any product that may be evaluated in this article, or claim that may be made by its manufacturer, is not guaranteed or endorsed by the publisher.
